# Upregulation of CREB1 by Mycobacterium Tuberculosis Transcriptionally Activates TREM2 to Promote Macrophage M2 Polarization

**DOI:** 10.1002/cbf.70284

**Published:** 2026-08-17

**Authors:** Yufeng Zhu, Mingzhong Xu, Danxia Liu, Wei Ning, Miaomiao Wang, Feifan Huang, Zhen An, Jingsong Qiu, Xian Tang, Peipei Wang, Bin Tian, Lan Wen

**Affiliations:** ^1^ Department of Clinical Laboratory Changsha Municipal Center for Disease Control and Prevention Changsha Hunan Province China; ^2^ Department of Clinical Laboratory The Central Hospital of Shaoyang Shaoyang Hunan Province China; ^3^ Department of Clinical Laboratory Yongzhou Municipal Center for Disease Control and Prevention Yongzhou Hunan Province China

**Keywords:** CREB1, M2 polarization, THP‐1 cells, TREM2

## Abstract

Tuberculosis, caused by Mycobacterium tuberculosis (MTB), affects approximately 25% of people globally as latent infection (LTBI). Although macrophage CREB activation promotes MTB survival, the underlying mechanisms remain unclear. This study reveals, for the first time, how MTB modulates M2 macrophage polarization through the CREB1/TREM2 signaling pathway. Mononuclear macrophages were isolated from clinical sample. Flow cytometry was used to determine the M2 polarization ratio. The expression of TNF‐α, IL‐10, and IL‐1β was determined by ELISA. The mRNA expression of iNOS, IL‐1β, CD206, Arg‐1, IL‐10, CREB1, and TREM2 was assessed by qPCR. The protein expression of CREB1, CD206, Arg‐1, IL‐10, and TREM2 was evaluated by western blot. The colony‐forming unit (CFU) assay was used to detect the survival of MTB. CHIP and Dual‐luciferase reporter assays were used to confirm the binding of CREB1 and TREM2. Clinical sample analysis revealed that the expression levels of CREB1 and TREM2 in peripheral blood mononuclear macrophages (PBMCs) were significantly upregulated in tuberculosis patients. Following H37Rv infection, an increase in the M2 macrophage proportion was observed. Infection with MTB also elevated the protein level of IL‐10, as well as CREB1 mRNA and protein expression. Transfecting sh‐CREB1 into macrophages or adding CREB inhibitors 666‐15 markedly reduced intracellular MTB CFU counts, implying a potential restrictive effect on viable MTB load within macrophages. Knockdown of TREM2 similarly decreased MTB CFU burden and restrained macrophage M2 polarization. Furthermore, transfecting sh‐CREB1 into macrophages can suppress the proliferation of MTB within macrophages, and these effects could be further counteracted by the action of oe‐TREM2. These results elucidated that MTB promoted M2 macrophage polarization through CREB1/TREM2.

## Summary

- MTB infection promotes macrophage M2 polarization and upregulates CREB1.
- Upregulated CREB1 transcriptionally activates TREM2 for promoting macrophage M2 polarization.

**Abbreviations**
Arg‐1: arginase‐1
CFU: colony‐forming unit
ChIP: chromatin immunoprecipitation
CREB1: cAMP response element binding protein 1
ELISA: enzyme‐linked immunosorbnent assay
IL‐10: interleukin‐10
IL‐1β: interleukin‐1β
iNOS: inducible nitric oxide synthase
LTBI: latent infection of mycobacterium tuberculosis
MTB: mycobacterium tuberculosis
qPCR: quantitative real‐time PCR
TLR4: toll‐like receptor 4
TNF‐α: tumor necrosis factor‐α
TREM2: triggering receptor expressed on myeloid cells 2

## 1 Introduction

Tuberculosis, a chronic infectious disease caused by Mycobacterium tuberculosis (MTB), is like a “reef” hidden within the defense line of human health, threatening the normal functioning of the body at all times [1, 2]. If left untreated, this condition may pose a threat to multiple organs throughout the body, with the lungs being the most frequently affected [3, 4]. Latent infection of Mycobacterium tuberculosis (LTBI) represents that the body is in the state of maintaining an immune response to the antigen stimulation of the bacterium, and there are no corresponding clinical and imaging characteristics of active tuberculosis [5, 6]. According to estimates from professional organizations, approximately 25% of the global population has been quietly invaded by MTB [7]. However, the immune mechanism of the host against Mycobacterium tuberculosis in LTBI remains unclear. Thus, an in‐depth study of the host's immune mechanisms against Mycobacterium tuberculosis is essential for understanding its pathogenesis.

cAMP response element‐binding protein (CREB), as a transcription factor, plays a crucial role in the survival, proliferation, and differentiation processes of cells [8, 9, 10]. Related studies have confirmed that once Mycobacterium tuberculosis invades human macrophages, it activates the CREB transcription factor, which creates favorable conditions for bacterial survival: it not only reduces the transport of NF‐kB to the nucleus but also limits phagolysosome fusion through inhibition of necrosis signaling [11]. In addition, knocking down CREB can effectively inhibit the MTP proliferation in RAW mouse macrophage line experiments. However, studies regarding CREB in human macrophages infected with Mycobacterium tuberculosis remain scant at present. The intricate molecular mechanisms underlying it are yet to be delved into further.

Triggering receptor expressed on myeloid cells 2 (TREM2), belonging to the TREM transmembrane glycoprotein family, is a type of receptor located on the cell surface [12, 13]. Multiple studies have shown that TREM2 plays the role of a non‐glycosylated mycolic acid receptor in mycobacteria, which can limit the anti‐mycobacterial activation process of macrophages [14]. Further research reveals that TREM2 plays a crucial role in the “game” between Mycobacterium tuberculosis and human macrophages [15]. When Mycobacterium tuberculosis invades macrophages, TREM2 is activated, which interferes with the normal immune recognition and killing process of macrophages. In addition, there is ample evidence to suggest that TREM2 significantly promotes macrophage polarization towards the M2 type [16]. The polarization state of macrophages is closely related to immune function. M2 polarized macrophages often exhibit unique immune regulatory characteristics, and TREM2 seems to be the key “catalyst” for this polarization process [16]. However, the mechanism of TREM2 involvement in MTB‐infected macrophages remains to be fully elucidated.

Using JASPAR, we made a prediction that CREB1 harbors a binding site within the promoter region of TREM2 [17]. Based on this finding, we have formulated a speculation: when macrophages are infected by MTB, the pathogen might boost its own survival and proliferation. This could be achieved by escalating the transcription of CREB1, which in turn activates TREM2, thus potentially tipping the balance of the host–pathogen interaction in favor of the mycobacterium.

In summary, we propose the hypothesis that upregulation of CREB1 transcription activates TREM2 after MTB infection of macrophages, promotes M2 polarization in macrophages, and promotes MTB cell survival and immune escape.

## 2 Methods

### 2.1 Isolation and Differentiation of Mononuclear Macrophage Cells

This retrospective study used leftover blood samples collected after routine diagnostic procedures. Peripheral blood (10–15 mL) was collected from healthy donors (*n* = 15) and patients with confirmed pulmonary tuberculosis who had not received antituberculosis treatment (*n* = 15) using heparin‑anticoagulant tubes. This study protocol was approved by the Ethics Committee of Changsha Municipal Center for Disease Control and Prevention (CSCDC2026‐EC‐034). The requirement for written informed consent was waived by the ethics committee because all specimens were fully de‐identified, the research involved minimal risk to participants, and it was practically infeasible to contact individual patients to obtain consent. All operations strictly complied with the principles of the Declaration of Helsinki and the BRISQ guidelines for human biospecimen reporting. Peripheral blood mononuclear cells (PBMCs) were isolated using Ficoll–Paque (GE Healthcare, GE17‑1440‑02) according to the manufacturer's instructions and a previous study [18]. Briefly, whole blood was centrifuged at 1600 rpm for 15 min to remove plasma. An equal volume of PBS and 10 mL of Ficoll–Paque solution were added, followed by centrifugation at 400 × *g* for 30 min at room temperature. The white mononuclear cell layer was aspirated into a new centrifuge tube, mixed with more than five volumes of PBS, and centrifuged at 250 × *g* for 10 min. The supernatant was discarded, and the washing step was repeated once. Finally, the cell pellet was resuspended in 2 mL of RPMI‑1640 medium (Gibco, USA) supplemented with 10% fetal bovine serum (Gibco, USA), adjusted to a density of 1 × 10^7^ cells/mL. After incubation at 37°C under 5% CO_2_ for 2 h, the supernatant was removed, and the adherent cells were gently washed twice with PBS. The purified monocyte‑derived macrophages were used directly for subsequent qPCR experiments.

### 2.2 Mycobacteria Culture

Mtb strain H37Rv (American Type Culture Collection, ATCC) was passaged routinely in Middlebrook 7H9 broth (BD Biosciences, USA) with 0.05% Tween‐80 (Merck KGaA, Germany) and BD microbiology systems (10% oleic acid albumin dextrose catalase enrichment, OADC) at 37°C. The growth of H37Rv was assessed by OD absorbance at 600 nm.

### 2.3 Cell Culture and Infection

The THP‐1 cells were obtained from Wuhan Procell company (China) and maintained in RPMI‐1640 medium (Hyclone, USA) with 10% Fetal Bovine Serum (FBS, Hyclone), 0.05 mM β‐mercaptoethanol (Yeasen Biotechnology, Shanghai, China), and 1% penicillin/streptomycin (P/S, Hyclone) at 37°C in 5% CO_2_. The cells were differentiated by incubating in RPMI‐1640 medium containing 50 ng/ml Phorbol‐12‐myristat‐13‐acetat (PMA, Merck KGaA), 10% FBS, and 1% P/S (Hyclone) for 24 h, and cultured in fresh medium for another 24 h. Differentiated cells were infected with Mtb strain H37Rv at a multiplicity of infection (MOI) of 10 for 4 h, then washed and incubated with medium supplemented with gentamycin for 2 h. After removing the adhering bacteria, the cells were ultimately incubated with DMEM containing 10% FBS (Hyclone) for the scheduled time periods.

### 2.4 Cell Transfection

The knockdown/overexpression plasmids (sh‐CREB1, oe‐CREB1, oe‐TREM2) and negative control plasmids (sh‐NC, oe‐NC) were synthesized by GenePharma Technologies (Shanghai, China) and transfected into the THP‐1 cells or differentiated THP‐1 cells according to the manufacturer's protocol using Lipofectamine 2000 reagent (Invitrogen, USA). The medium of transfected cells was replaced after 4 h, and the cells were cultured prior to analysis. The shRNA sequence of CREB1 was displayed in Table 1.

**Table 1:** The target sequence of sh‐CREB1.

Gene	Target sequences
sh‐CREB1	ACGGTGCCAACTCCAATTTAC
sh‐NC	GGAAGTGCCTGTTCCAGCAATG

### 2.5 Flow Cytometry

The THP‐1 cells treated with different interventions were harvested and washed with cold phosphate‐buffered saline (PBS) three times, then the cells were resuspended and stained with PE anti‐human CD206 (Biolegend, 321105, USA) for 30 min at 4°C in the dark. After washing with cold PBS, the cells were detected and quantified using a FACSCanto II flow cytometer (BD Biosciences) and FlowJo software (BD Biosciences).

### 2.6 Bacterial Proliferation Assays

The colony‐forming unit (CFU) assay was used to quantify intracellular viable MTB colony counts following its infection of THP‐1 cells that were treated with PMA and transfected with different plasmids.

### 2.7 ELISA Assays

The levels of TNF‐α, IL‐10, and IL‐1β in the culture supernatant of PMA‐treated THP‐1 cells were measured by ELISA kits (Beyotime Biotechnology, PT518, PI528, PI305, Shanghai, China). The cell culture supernatant was collected after the cells were infected with H37Rv, and measurements were performed according to the instructions of the ELISA kits. Eventually, a microplate reader (BioTek, USA) was used to detect the density at 450 nm of each sample.

### 2.8 Quantitative Real‐Time Polymerase Chain Reaction (qPCR)

Total RNA was extracted from THP‐1 cells to be tested by using TRIZOL solution (Invitrogen) and reverse‐transcribed into cDNA using the PrimeScript RT Kit (TAKARA, Japan). Expression of related genes was detected by qPCR using Power SYBR Green PCR Master Mix (Thermo Scientific) and CFX Opus 96 Real‐Time PCR System (Bio‐Rad, USA), in line with the manufacturer's instructions. GAPDH was selected as a housekeeping gene to normalize the data using the 2^−ΔΔCt^ method. The qPCR protocol consists of an initial denaturation at 95°C for 3 min (1 cycle), followed by 40–45 cycles of amplification (95°C for 10 s, primer‐specific annealing at 55°C–65°C for 20 s, and extension at 72°C for 20 s, if required).

The specific primer sequences are shown in Table 2.

**Table 2:** The primer sequence list used for qPCR.

Gene	Forward primer (5ʹ−3ʹ)	Reverse primer (5ʹ−3ʹ)
CREB1	TGCAACATCATCTGCTCCCA	CTGAATAACTGATGGCTGGGC
CD206	TCCGGGTGCTGTTCTCCTA	CCAGTCTGTTTTTGATGGCACT
iNOS	CCAGCTAGCCAAAGTCACCAT	GTCTCGGAGCCATACAGGATT
IL‐1β	ATGATGGCTTATTACAGTGGCAA	GTCGGAGATTCGTAGCTGGA
Arg‐1	GTGGAAACTTGCATGGACAAC	AATCCTGGCACATCGGGAATC
IL‐10	GACTTTAAGGGTTACCTGGGTTG	TCACATGCGCCTTGATGTCTG
TREM2	GGTCAGCACGCACAACTTG	CGCAGCGTAATGGTGAGAGT
GAPDH	GGAGTCCACTGGCGTCTTCA	GTCATGAGTCCTTCCACGATACC

### 2.9 Western Blot Assay

The THP‐1 cell samples to be detect were harvested, washed and homogenized by RIPA lysis buffer (Beyotime Biotechnology) on ice. After quantifying by BCA kits (Beyotime Biotechnology), equal amounts of samples were separated by SDS‐PAGE and transferred to PVDF membranes (Merck Millipore, USA). Thereafter, the membranes were blocked with 5% skim milk (Yeasen Biotechnology) and incubated sequentially with the antibodies in Table 3. The primary antibodies were incubated overnight at 4°C, then the second antibody was incubated for 2 h at room temperature after washing three times with TBST buffer. Finally, the protein blots were imaged using the chemiluminescent (ECL) reagent (Thermo Scientific) and a ChemiDoc MP system (Bio‐Rad). The images obtained were further analyzed using ImageJ software (National Institutes of Health, USA).

**Table 3:** Primary antibodies used in western blotting assay.

Antibody	Company (Cat. No.)	Working dilution
CREB1	Cell signaling technology (#9197)	1:1000
CD206	Cell signaling technology (#91992)	1:1000
Arg‐1	Cell signaling technology (#93668)	1:1000
IL‐10	Abcam (ab133575)	1:1000
TREM2	Abcam (ab318262)	1:1000
Goat Anti‐Rabbit IgG H&L (HRP)	Abcam (ab97051)	1:10,000

### 2.10 Chromatin Immunoprecipitation (ChIP)

The ChIP assay was performed using the simple ChIP Enzymatic ChIP Kit (Cell Signaling Technologies, USA). Briefly, the THP‐1 cells were successively incubated with 1% formaldehyde for 10 min and glycine for 5 min at room temperature. After the DNA was fractured on the basis of the manufacturer's instructions, the chromatin complex was incubated respectively with negative control IgG (1:100, ab171870, Abcam) and CREB1 antibody (1:50, #9197, Cell signaling technology). Subsequently, the immunoprecipitated promoter regions of TREM2 were assessed by qPCR following de‐crosslinking with NaCl and proteinase K. qPCR primers were designed according to the binding sites of CREB1 with TREM2 promoter regions predicted using the JASPAR database (https://jaspar.elixir.no/).

### 2.11 Dual‐Luciferase Reporter Assay

The interaction between the CREB1 and TREM2 was assessed by dual‐luciferase reporter assays. Briefly, the potential binding and mutant of TREM2 promoter sequences were cloned into the pGL3 vector (GenePharma Technologies), that is, TREM2‐WT/MUT. Subsequently, the above vectors were respectively co‐transfected with sh‐NC and sh‐CREB1 using Lipofectamine 2000 reagent (Invitrogen). The Dual‐Glo luciferase assay system (Promega, USA) was applied to detect the luciferase activity after transfection for 48 h on a microplate reader (BioTek) in accordance with the manufacturer's protocol. The experiment was carried out in three replicates.

### 2.12 Statistical Analysis

All experimental data were displayed as mean ± standard deviation (SD) and analyzed using GraphPad Prism Version 8.0 software (GraphPad Prism Software, USA). The comparison of two groups was performed by a two‐tailed paired Student's *t*‐test, and the differences were regarded as significant when the *p*‐value was less than 0.05.

## 3 Results

### 3.1 MTB Infection Promotes Macrophage M2 Polarization and Upregulates CREB1

**Figure 1:**
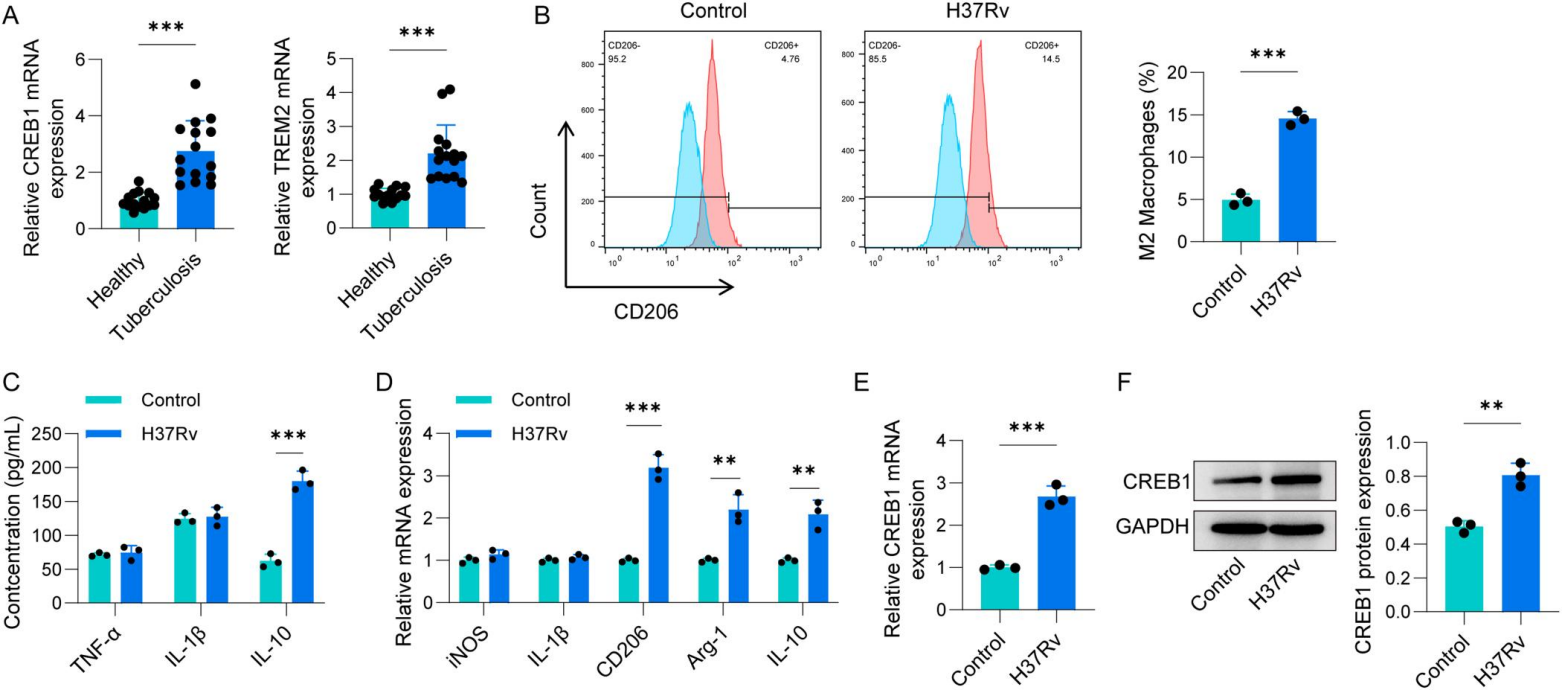
MTB infection promotes macrophage M2 polarization and upregulates CREB1. (A) The mRNA expression of CREB1 and TREM2 in peripheral blood mononuclear macrophages was determined by qPCR (*n* = 15). (B) M2 macrophage ratio after H37Rv infection was measured by flow cytometry. (C) The levels of TNF‐α, IL‐10 and IL‐1β in cell culture supernatant were detected by ELISA. (D) qPCR analysis of mRNA expression of M1 markers (iNOS, IL‐1β) and M2 markers (CD206, Arg‐1, IL‐10) in macrophages with or without MTB infection. (E, F) The mRNA and protein expression levels of CREB1 in macrophages upon MTB infection determined by qPCR and Western blot. Data were displayed as mean ± SD. *n* = 3, **p < 0.01, ***p < 0.001.

We first evaluated the effect on the M0 macrophage phenotype after combined Mycobacterium tuberculosis infection. Clinical sample analysis revealed that the expression levels of CREB1 and TREM2 in PBMCs were significantly upregulated in tuberculosis patients compared with healthy volunteers (Figure 1A). Furthermore, it was discovered that the proportion of M2 increased following H37Rv infection (Figure 1B). Subsequently, the level of cytokines was quantified by means of ELISA. Upon MTB infection, no significant fluctuations were detected in the levels of TNF‐α and IL‐1β, yet the concentration of IL‐10 underwent a notable increase (Figure 1C). In addition, we evaluated the expression of different genes subjected to the injection of Mycobacterium tuberculosis. Obviously, the levels of iNOS and IL‐1β remained unchanged after infection, while the levels of Arg‐1, CD206, and IL‐10 increased (Figure 1D). Moreover, both mRNA and protein expression of CREB1 increased after MTB infection (Figure 1E, F). These findings indicate that MTB infection directly promotes an M2‐like phenotype from an M0 starting point and upregulates CREB1.

### 3.2 Knockdown of CREB1 Reduces Intracellular MTB CFU Burden and Restrains Macrophage M2 Polarization

**Figure 2:**
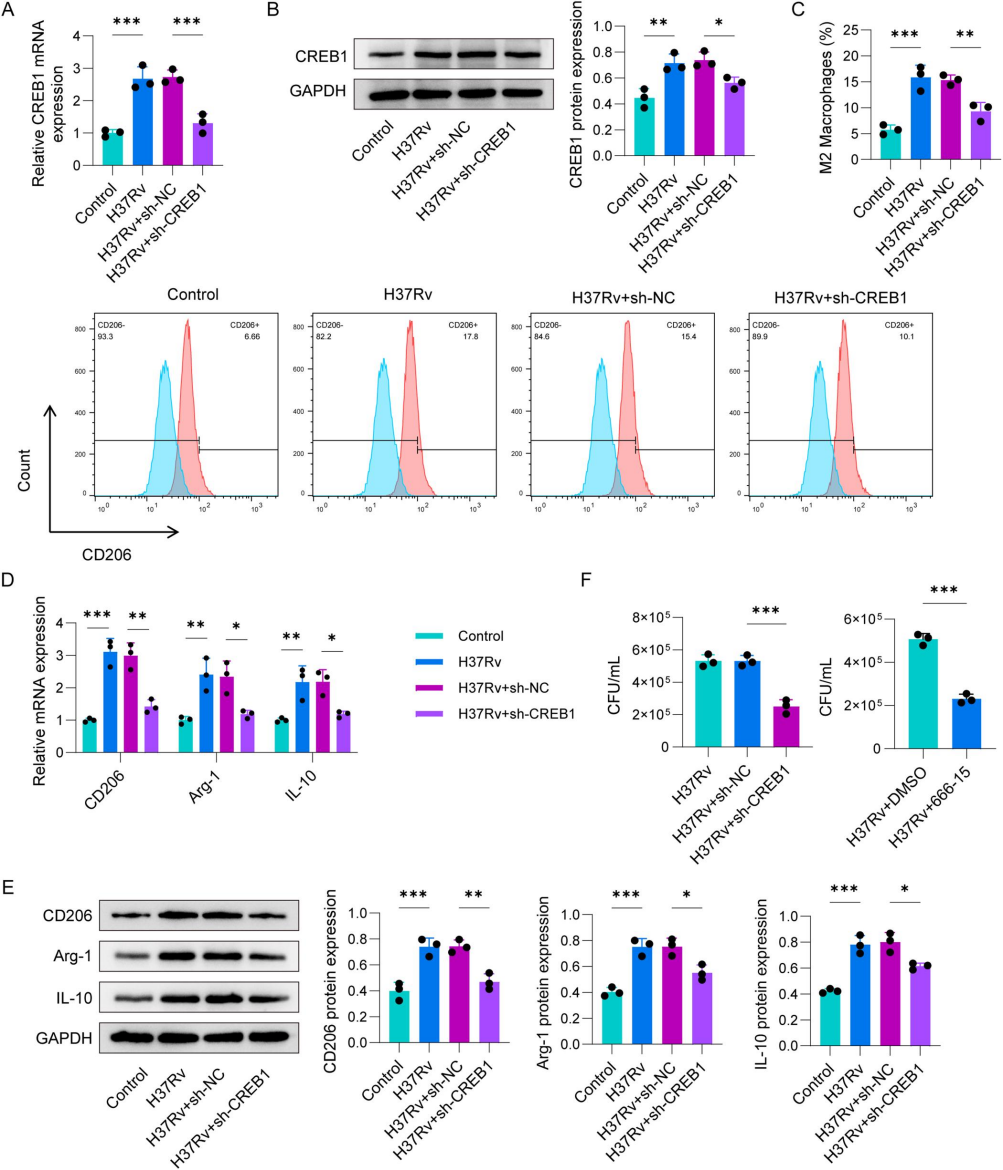
Knockdown of CREB1 reduces intracellular MTB CFU burden and restrains macrophage M2 polarization. The mRNA (A) and protein (B) expression of CREB1 in macrophages after transfection with sh‐CREB1 or sh‐NC upon MTB infection were determined by western blot and qPCR, respectively. (C) M2 macrophage ratio in different treatment groups was measured by flow cytometry. (D, E) qPCR and Western blot was used to examine the mRNA and protein levels of M2 markers (CD206, Arg‐1, IL‐10) in each group. (F) CFU assay detected the intracellular burdens of MTB in macrophages pretreated with sh‐NC, sh‐CREB1, CREB1 inhibitor 666‐15 or DMSO. Data were displayed as mean ± SD. *n* = 3, *p < 0.05, **p < 0.01, ***p < 0.001.

To further verify the regulatory effect of CREB1 on intracellular MTB viable load and macrophage polarization, sh‐CREB1 and sh‐NC were transfected into cells, respectively. Both mRNA and protein expression of CREB1 levels were simultaneously elevated after MTB infection and significantly decreased when sh‐CREB1 was added (Figure 2A,B). Consistently, the proportion of M2 cells rose following MTB infection and dropped after CREB1 knockdown (Figure 2C). Subsequently, we measured the impact of sh‐CREB1 on the mRNA and protein expression of CD206, Arg‐1, and IL‐10. Elevated levels of Arg‐1, CD206, and IL‐10 caused by MTB infection, were reversed after CREB1 knockdown (Figure 2D,E). Furthermore, previous studies have demonstrated that the inhibition of CREB1 could boost macrophages' ability to regulate the growth of Mycobacterium tuberculosis. Thus, Macrophages were pretreated with sh‐NC, sh‐CREB1, CREB inhibitors 666‐15, or DMSO prior to MTB infection. It was observed that either transfecting sh‐CREB1 into macrophages or adding CREB inhibitors 666‐15 significantly reduced intracellular MTB CFU counts, suggesting a potential limitation on viable MTB burden within macrophages (Figure 2F). These results indicate that CREB1 knockdown suppresses macrophage M2 polarization and is accompanied by lower intracellular MTB CFU counts.

### 3.3 CREB1 Transcriptionally Activates TREM2

**Figure 3:**
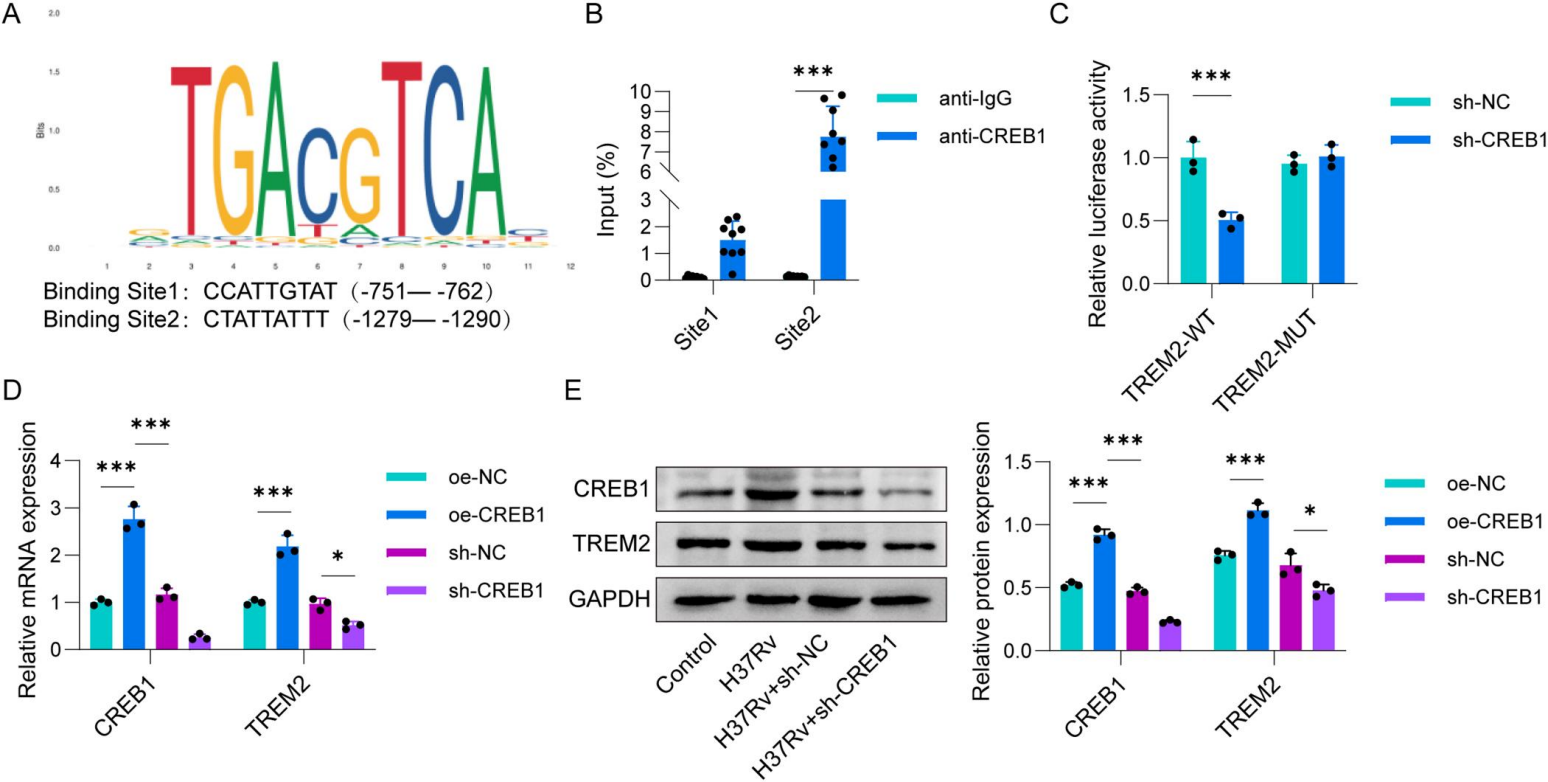
CREB1 directly binds to the TREM2 promoter and transcriptionally activates TREM2 expression. (A) JASPAR database predicted potential CREB1 binding sites in the TREM2 promoter region. (B) ChIP‐qPCR assay verified the binding enrichment of CREB1 to the TREM2 promoter. (C) Dual‐luciferase reporter assay confirmed the transcriptional binding effect between CREB1 and TREM2 promoter. (D, E) qPCR and Western blot detected the mRNA and protein expression of CREB1 and TREM2 after CREB1 knockdown or overexpression. Data were displayed as mean ± SD. *n* = 3, *p < 0.05, ***p < 0.001.

To gain deeper insights into the functional mechanisms of CREB1 within macrophages, we initially resorted to the JASPAR database to forecast the binding sites of CREB1. It was discovered that CREB1 has two high‐scoring binding sites in the TREM2 promoter region (Figure 3A). Subsequently, we carried out chromatin immunoprecipitation (ChIP) assays, and the results indicated that there were detectable enrichment levels of TREM2 at site 2 (Figure 3B). Furthermore, the dual luciferase reporter gene method confirms the binding of CREB1 and TREM2. It was observed that knocking down CREB1 significantly reduced luciferase activity in WT TREM2, but the TREM2 mutation was not affected (Figure 3C). Moreover, overexpression of CREB1 significantly boosted the mRNA and protein levels of CREB1 and TREM2. Conversely, after knocking down CREB1, the levels of both dropped significantly (Figure 3D,E). Thus, functioning as a downstream factor of CREB1, TREM2 serves to promote its expression upon the activation of CREB1.

### 3.4 Knockdown of TREM2 Decreases Intracellular MTB CFU Burden and Suppresses Macrophage M2 Polarization

**Figure 4:**
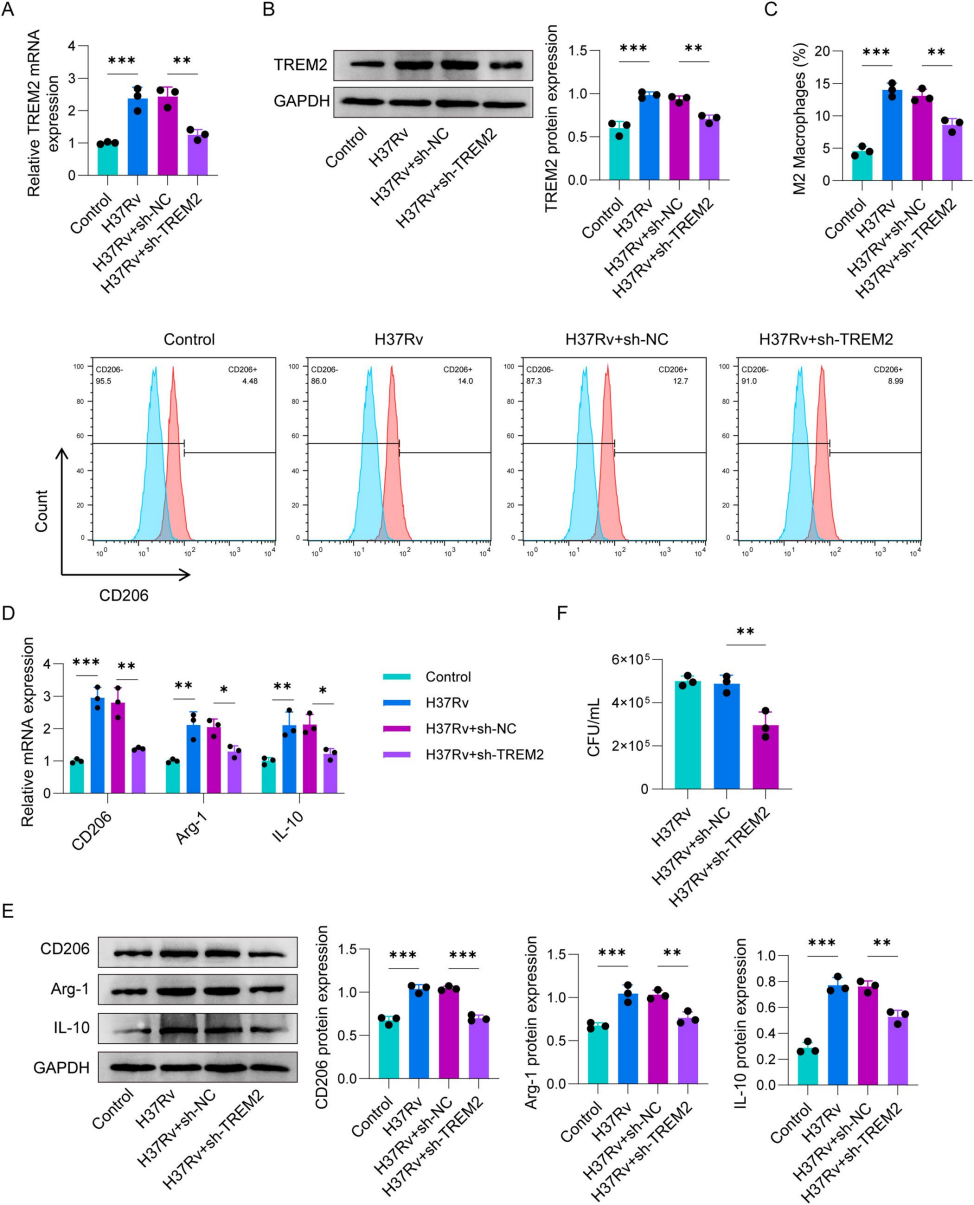
Knockdown of TREM2 decreases intracellular MTB CFU burden and suppresses macrophage M2 polarization. (A, B) qPCR and Western blot analysis of TREM2 mRNA and protein expression in macrophages transfected with sh‐TREM2 or sh‐NC under MTB infection. (C) Flow cytometry determined the percentage of M2‐polarized macrophages in different groups. (D, E) qPCR and Western blot detected the mRNA and protein levels of M2 phenotypic markers (CD206, Arg‐1, IL‐10) after TREM2 silencing. (F) CFU assay evaluated the intracellular burdens of MTB in TREM2‐knockdown macrophages. Data were displayed as mean ± SD. *n* = 3, *p < 0.05, **p < 0.01, ***p < 0.001.

In order to validate the regulatory role of TREM2 on intracellular MTB viable load and macrophage polarization, sh‐TREM2 and sh‐NC were transfected into cells, respectively. Apparently, both mRNA and protein expression of TREM2 were up‐regulated following MTB infection, and significantly counteracted when sh‐TREM2 was added (Figure 4A,B). In addition, the proportion of M2 cells rose following MTB infection and dropped after TREM2 knockdown, which was similar to CREB1 silencing (Figure 4C). Subsequently, we measured the impact of sh‐TREM2 on the mRNA and protein expression of CD206, Arg‐1, and IL‐10. It was demonstrated that MTB infection leads to an increase in Arg‐1, CD206, and IL‐10 levels, which are reversed after TREM2 gene knockout (Figure 4D,E). Furthermore, it was observed that transfecting sh‐TREM2 into macrophages markedly lowered intracellular MTB CFU numbers (Figure 4F). These results indicate that TREM2 knockdown decreases intracellular MTB CFU burden and macrophage M2 polarization.

### 3.5 MTB Promotes Macrophage M2 Polarization through CREB1/TREM2

**Figure 5:**
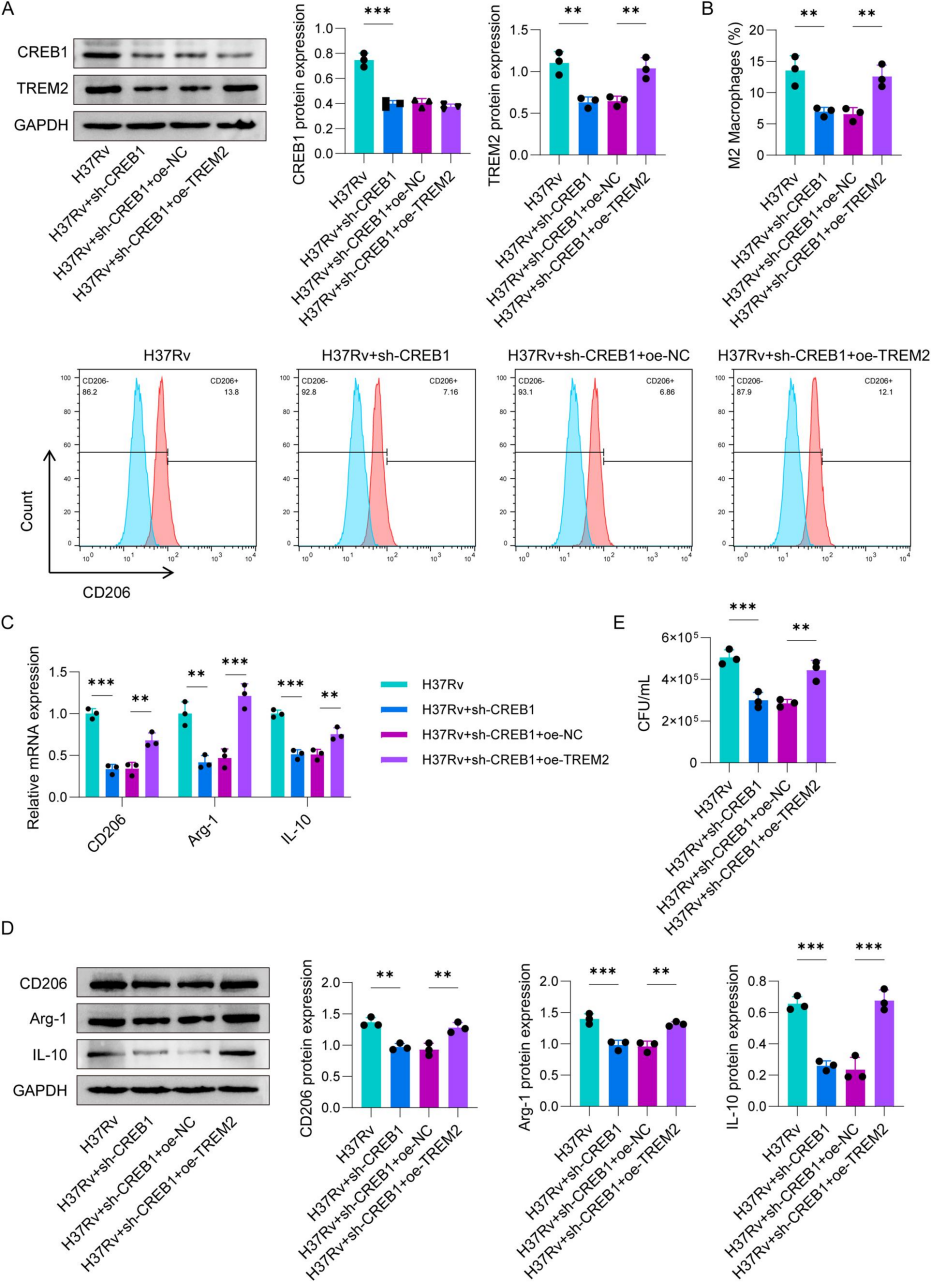
MTB induces macrophage M2 polarization via the CREB1/TREM2 signaling axis. (A) Western blot detected the protein expression of CREB1 and TREM2 in macrophages transfected with sh‐CREB1 alone or co‐transfected with oe‐TREM2. (B) Flow cytometry analysis of M2 macrophage proportion in different rescue experimental groups. (C, D) qPCR and Western blot examined the mRNA and protein levels of M2 markers (CD206, Arg‐1, IL‐10) in the rescue assay. (E) CFU assay detected intracellular MTB burdens in macrophages of different treatment groups. Data were displayed as mean ± SD. *n* = 3, **p < 0.01, ***p < 0.001.

In the previous experiment, we demonstrated that knocking down CREB1 and TREM2 can inhibit MTB infection and macrophage M2 polarization. To further confirm their role in inhibiting MTB infection and macrophage M2 polarization, we transfected sh‐CREB1, sh‐CREB1 with oe‐NC, and sh‐CREB1 with oe‐TREM2 into macrophages, respectively. It was elucidated by the western blot that knocking down CREB1 led to a decline in the protein levels of CREB1 and TREM2. In contrast, overexpressing TREM2 triggered an elevation in its corresponding levels under the treatment of sh‐CREB1 (Figure 5A). Moreover, the knockdown of CREB1 results in a reduction in the proportion of M2 macrophages, whereas the overexpression of TREM2 brings about an increase (Figure 5B). Consistently, knockdown of CREB1 led to a dramatic decrease in Arg‐1, CD206, and IL‐10 levels, while overexpression of TREM2 reversed these levels (Figure 5C,D). Eventually, it was observed that transfecting sh‐CREB1 into macrophages can suppress the proliferation of MTB within macrophages, and these effects could be further counteracted by the action of oe‐TREM2 (Figure 5E). These results demonstrate that MTB promotes macrophage M2 polarization through CREB1/TREM2.

## 4 Discussion

Mycobacterium tuberculosis is the causative agent of tuberculosis, which has puzzled human beings for thousands of years. The key to its ability to infect new hosts and spread widely lies in a complex strategy of evading host immune surveillance, as well as utilizing certain immune mechanisms for its own benefit [19, 20, 21]. Upon infection of the human body by Mycobacterium tuberculosis, the immune system is triggered. Subsequently, immune cells, encompassing both innate immune cells and adaptive immune cells, experience a sequence of intricate and well‐ordered alterations [22, 23, 24]. In our study, we systematically elucidated that MTB promoted macrophage M2 polarization through the CREB1/TREM2 signaling axis.

Macrophages, as the main host cells against MTB, play a vital role in the fight against tuberculosis infection [25]. Mycobacterium tuberculosis affects macrophages through various mechanisms, thereby inhibiting their bactericidal functions and promoting their own survival, leading to chronic infection and immune evasion. Existing evidence shows that Mycobacterium tuberculosis can also induce macrophages to polarize towards M1 or M2 type [26, 27]. M1 exerts inhibitory effects on the growth of MTB, while M2 exerts anti‐inflammatory effects and mediates immune escape [28, 29]. Indeed, Mycobacterium tuberculosis can induce macrophages to produce M2 phenotype during the latent period of tuberculosis infection, thereby improving their survival rate [30]. Herein, we found that macrophages promote M2 polarization and correlate with altered viable MTB loads in host cells by upregulating CREB1. Our study identified an upregulation of CREB in tuberculosis patients, which aligns with its recognized function as a positive transcriptional regulator of gamma interferon during latent tuberculosis infection [31]. In addition, the activation of CREB in macrophages would enhance the viability of bacteria [11]. However, the relationship between CREB expression level and macrophage polarization remains elusive.

Furthermore, we investigated the molecular mechanism of how the upregulation of CREB affects macrophage M2 polarization. Recently, much progress has been made in exploring M2 polarization of macrophages caused by Mycobacterium tuberculosis infection [30]. Rv1987 protein, which was secreted by Mycobacterium tuberculosis, could induce M2 polarization of macrophages by activating the PI3K/Akt1/mTOR signaling pathway [32]. In addition, Mycobacterium tuberculosis heparin‐binding hemagglutinin drives macrophage M2 polarization through TLR4 [33]. In this study, we have determined that TREM2 serves as the downstream target of CREB1. It was well‐established that TREM2 facilitated the uptake of Mycobacterium tuberculosis by macrophages. It acts as a key regulator, effectively blocking the production of TNF‐α, IL‐1β, reactive oxygen species, and IL‐10, which is consistent with our research [34]. Moreover, TREM2 promotes macrophage polarization, which was found in various diseases, including osteoarthritis, pulmonary fibrosis, and lung ischemia‐reperfusion injury [16, 35, 36, 37]. Consistently, knockdown of CREB1 results in a reduction in the proportion of M2 macrophages, which can be counteracted by the overexpression of TREM2. These results demonstrated that MTB promotes macrophage M2 polarization through CREB1/TREM2.

In this study, we investigated for the first time how Mycobacterium tuberculosis affects macrophage M2 polarization via CREB1/TREM2 signaling cascades. Consistently, previous studies revealed that TREM2 activation by COG1410 alleviated neural damage through the activation of Akt/CREB/BDNF signaling axis [38]. Despite these established observations, the present study provides several distinct insights. First, although CREB1 activation is known, we demonstrate for the first time that CREB1 functions directly upstream of TREM2 to drive M2 polarization, thereby connecting these two previously separate pathways. Second, previous studies on TREM2 have largely focused on its role in neurodegenerative diseases, metabolic Diseases and cancer; our work extends its functional relevance to infectious settings, specifically MTB infection. Nevertheless, several limitations of the present study should be acknowledged. First, all mechanistic explorations rely heavily on the PMA‐differentiated THP‐1 macrophage model, which cannot fully recapitulate the biological characteristics of human primary monocyte‐derived macrophages. Second, this study is limited to in vitro cellular experiments and clinical peripheral blood sample detection; we lack rigorous in vivo animal model validation. Thus, additional research is warranted to further understand the functional roles of CREB1/TREM2 in tuberculosis.

## 5 Conclusions

Herein, we elucidated that upregulation of CREB1 by Mycobacterium tuberculosis transcriptionally activates TREM2 for promoting macrophage M2 polarization in vitro. This study raised the possibility of treating tuberculosis by targeting CREB1/TREM2 cascades.

## Author Contributions

Lan Wen guaranteed the integrity of the entire study. Yufeng Zhu, Mingzhong Xu, and Miaomiao Wang designed the study and literature research. Danxia Liu Wei Ning defined the intellectual content. Feifan Huang and Zhen An performed the experiments. Jingsong Qiu collected the data. Xian Tang, Peipei Wang, and Bin Tian analyzed the data. Yufeng Zhu and Mingzhong Xu wrote the main manuscript and prepared figures. All authors reviewed the manuscript.

## Ethics Statement

This study protocol was approved by the Ethics Committee of Changsha Municipal Center for Disease Control and Prevention (CSCDC2026‐EC‐034).

## Consent

The authors have nothing to report.

## Conflicts of Interest

The authors declare no conflicts of interest.

## Code Availability

The authors have nothing to report.

## Declaration of Generative AI Use

During the preparation of this work the authors did not use any AI‐assisted technology.

## Data Availability

The data that support the findings of this study are available from the corresponding author upon reasonable request.
